# The potential role and mechanism of circRNA/miRNA axis in cholesterol synthesis

**DOI:** 10.7150/ijbs.84994

**Published:** 2023-05-29

**Authors:** Wujun Chen, Jiazhen Xu, Yudong Wu, Bing Liang, Mingzhe Yan, Chuandong Sun, Dong Wang, Xiaokun Hu, Li Liu, Wenchao Hu, Yingchun Shao, Dongming Xing

**Affiliations:** 1 Cancer Institute, The Affiliated Hospital of Qingdao University, Qingdao University, Qingdao Cancer Institute, Qingdao, Shandong, 266000, China.; 2 Department of Liver Disease Center, The Affiliated Hospital of Qingdao University, Qingdao, Shandong, 266000, China.; 3 Interventional Medicine Center, The Affiliated Hospital of Qingdao University, Qingdao, Shandong, 266000, China.; 4 Department of Community Health Promotion, Qingdao Municipal Center for Disease Control & Prevention, Qingdao Institute of Preventive Medicine, Qingdao, Shandong, 266033, China.; 5 Department of Endocrinology, Qilu Hospital (Qingdao), Cheeloo College of Medicine, Shandong University, Qingdao, Shandong, 266000, China.; 6 School of Life Sciences, Tsinghua University, Beijing, 100084, China.

**Keywords:** Cholesterol synthesis, circRNAs, HMGCR, SQLE, miR-122, nucleic acid drugs.

## Abstract

Cholesterol levels are an initiating risk factor for atherosclerosis. Many genes play a central role in cholesterol synthesis, including HMGCR, SQLE, HMGCS1, FDFT1, LSS, MVK, PMK, MVD, FDPS, CYP51, TM7SF2, LBR, MSMO1, NSDHL, HSD17B7, DHCR24, EBP, SC5D, DHCR7, IDI1/2. Especially, HMGCR, SQLE, FDFT1, LSS, FDPS, CYP51, and EBP are promising therapeutic targets for drug development due to many drugs have been approved and entered into clinical research by targeting these genes. However, new targets and drugs still need to be discovered. Interestingly, many small nucleic acid drugs and vaccines were approved for the market, including Inclisiran, Patisiran, Inotersen, Givosiran, Lumasiran, Nusinersen, Volanesorsen, Eteplirsen, Golodirsen, Viltolarsen, Casimersen, Elasomeran, Tozinameran. However, these agents are all linear RNA agents. Circular RNAs (circRNAs) may have longer half-lives, higher stability, lower immunogenicity, lower production costs, and higher delivery efficiency than these agents due to their covalently closed structures. CircRNA agents are developed by several companies, including Orna Therapeutics, Laronde, and CirCode, Therorna. Many studies have shown that circRNAs regulate cholesterol synthesis by regulating HMGCR, SQLE, HMGCS1, ACS, YWHAG, PTEN, DHCR24, SREBP-2, and PMK expression. MiRNAs are essential for circRNA-mediated cholesterol biosynthesis. Notable, the phase II trial for inhibiting miR-122 with nucleic acid drugs has been completed. Suppressing HMGCR, SQLE, and miR-122 with circRNA_ABCA1, circ-PRKCH, circEZH2, circRNA-SCAP, and circFOXO3 are the promising therapeutic target for drug development, specifically the circFOXO3. This review focuses on the role and mechanism of the circRNA/miRNA axis in cholesterol synthesis in the hope of providing knowledge to identify new targets.

## Introduction

Cholesterol is an important component of vertebrate organisms' membrane and plasma lipoproteins and regulates membrane fluidity and permeability. Cholesterol is also a precursor of steroid hormones, bile acids, and vitamin D. However, plasma cholesterol levels have been firmly the initiating factor of atherosclerosis, cardiovascular disease (ASCVD), and cancer, which are the leading causes of disease and death worldwide. Therefore, controlling cholesterol levels is essential for preventing and treating atherosclerosis 1-3. The human body gets 300-500 mg of cholesterol from the diet every day and produces about 700-900 mg of cholesterol from scratch 4. Approximately 50% of endogenous cholesterol is synthesized in the liver. HMG-CoA reductase (HMGCR) is the rate-limiting enzyme in cholesterol synthesis. Statins, which are the HMGCR inhibitors, have been widely used for the treatment of ASCVD. Statins also increase survival rates for cancer patients. However, the efficacy of statins was limited by compensatory increases in HMGCR protein. Statins also induced myopathy and hepatotoxicity 5, 6. Therefore, more research is required to identify new therapeutic targets and agents. Indeed, many genes play a key role in cholesterol synthesis, including HMG-CoA synthetase 1 (HMGCS1), mevalonate kinase (MVK), phosphomevalonate kinase (PMK), and mevalonate diphosphate decarboxylase (MVD, also named MDD), Farnesyl diphosphate farnesyl transferase 1 (FDFT1), squalene epoxidase (SQLE, also known as squalene monooxygenase (SM)), lanosterol synthase (LSS, also known as oxidosqualene cyclase (OSC)), farnesyl diphosphate synthase (FDPS, also named farnesyl pyrophosphate synthase (FPPS)), sterol 14alpha-demethylase (CYP51, also named cytochrome P450 family 51 subfamily A member 1 (CYP51A1)), transmembrane 7 superfamily member 2 (TM7SF2), lamin B receptor (LBR), methylsterol monooxygenase 1 (MSMO1), NAD(P) dependent steroid dehydrogenase-like (NSDHL), hydroxysteroid 17-beta dehydrogenase 7 (HSD17B7), 24-dehydrocholesterol reductase reductase (DHCR24, also known as seladin-1), cholestenol delta-isomerase (EBP), delta7-sterol 5-desaturase (SC5D), 7-Dehydrocholesterol reductase (DHCR7), isopentenyl diphosphate isomerase 1 and 2 (IDI1/2) 5, 7, 8. However, more studies are still needed to identify the medicinal properties of these targets.

Circular RNAs (circRNAs) are covalently closed-loop single-stranded RNA. CircRNAs have no 5'-3' polarities and a polyadenylated tail, making them much more stable and resistant to RNase R degradation than linear RNA. CircRNAs regulate gene expression by serving as the miRNA sponges, protein scaffolds and sponges, encoding proteins, and regulating splicing and transcription 9-11. So far, many small nucleic acid drugs and vaccines were approved for market, including Inclisiran (Proprotein convertase subtilisin/kexin-9 (PCSK9) siRNA), Patisiran (transthyretin (TTR) siRNA), Inotersen (TTR antisense oligonucleotide (ASO)), Givosiran (aminolevulinate synthase 1 (ALAS1) siRNA), Lumasiran (hydroxyacid oxidase 1 (HAO1) siRNA), Nusinersen (exon 7 of survival motor neuron 2 (SMN2) ASO), Volanesorsen (Apolipoprotein C3 (APOC3) ASO), Eteplirsen (exon 51 of Duchenne muscular dystrophy (DMD) ASO), Golodirsen (exon 53 of DMD ASO), Viltolarsen (exon 53 of DMD ASO), Casimersen (exon 7 of DMD ASO), Elasomeran (COVID19 Spike glycoprotein mRNA vaccine, also named mRNA-1273), Tozinameran (COVID19 Spike glycoprotein mRNA vaccine, also named BNT162b) 12-23. There are also multiple nucleic acid agents in preclinical or clinical studies. However, most of these agents are linear RNA drugs. Compared to linear RNA agents, circRNAs may have prolonged half-lives, high stability, low immunogenicity, low production cost, and high delivery efficiency due to the covalently closed structures. CircRNA agents are being developed by several companies, such as Orna Therapeutics, Laronde, CirCode, and Therorna 24, 25. Interestingly, circRNAs also regulated cholesterol synthesis by serving as miRNA sponges 26, 27. The formation, classification, and function of circRNAs and miRNAs, please see reviews by other groups 28, 29. Therefore, we focused on the role and mechanism of the circRNA/miRNA axis in regulating cholesterol synthesis to affect atherosclerosis and provided some potential targets for the diagnosis and treatment of atherosclerosis.

## The mechanism of cholesterol synthesis

**Figure 1:**
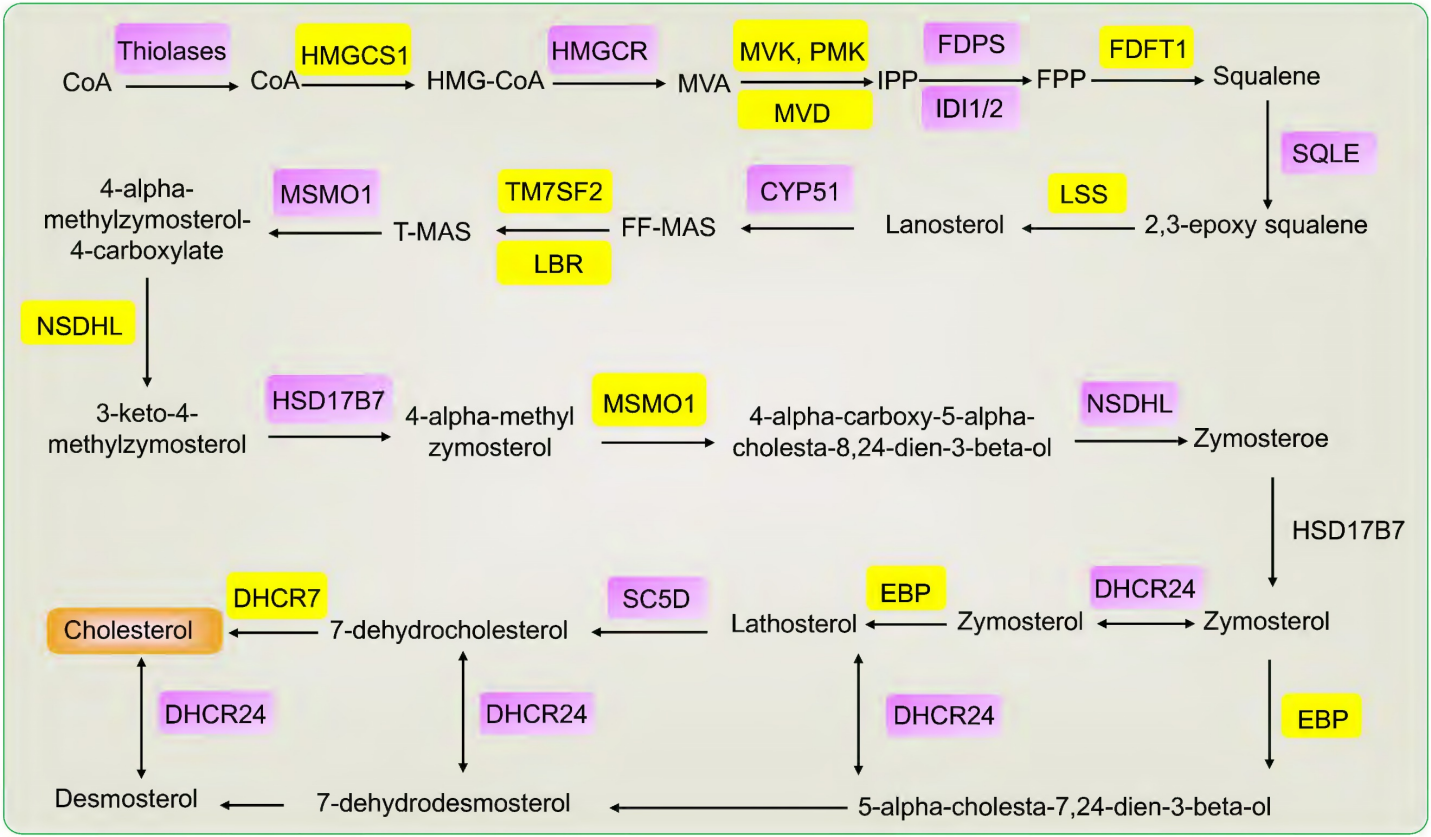
The genes and products of cholesterol biosynthesis pathway.

Cholesterol is biosynthesized in three main steps. Firstly, the synthesis of isoprene pyrophosphate (IPP). Acetyl-coenzyme A (CoA) is catalyzed to acetyl-CoA by thiolases and then catalyzed to form 3-hydroxy-3-methylglutaryl CoA (HMG-CoA) by HMGCS1. HMGCR catalyzes HMG-CoA to form mevalonate (MVA). Mevalonate was phosphorylated and decarboxylated to produce IPP by three sequential ATP-dependent Enzymes, including MVK, PMK, and MVD. Secondly, the synthesis of squalene. IPP is catalyzed to form farnesyl pyrophosphate (FPP) and then catalyzed to form squalene by FDFT1. Thirdly, the synthesis of cholesterol. Squalene is catalyzed to form 2,3-epoxy squalene by SQLE and then to form lanosterol by LSS. Lanosterol is catalyzed to form desmosterol and cholesterol after methyl transfer, oxidation, and decarboxylation reaction and then catalyzed to form cholesterol 7, 30, 31. According to KEGG, many genes are involved in cholesterol synthesis, such as FDPS, CYP51, TM7SF2, LBR, MSMO1, NSDHL, HSD17B7, DHCR24, EBP, SC5D, DHCR7, IDI1/2 8. Specifically, FDPS catalyzes the conversion of isopentenyl diphosphate into farnesyl pyrophosphate. CYP51 is a housekeeping gene of the cytochrome P450 that catalyzes the conversion of lanosterol into 4,4-dimethyl-5-alpha-cholesta-8,14,24-trien-3-beta-ol (FF-MAS). TM7SF2 encodes beta-hydroxysterol Delta (14)-reductase (C14SR, DHCR14) that catalyzes the conversion of FF-MAS into 14-demethyllanosterol (T-MAS). LBR and DHCR14 uniquely share the same delta-14 reductase activity in cholesterol biosynthesis. MSMO1 is an intermediate enzyme of cholesterol biosynthesis. NSDHL is a 3beta-hydroxysterol dehydrogenase that catalyzes the conversion of 4-alpha-carboxy-5-alpha-cholesta-8,24-dien-3-beta-ol into zymosterone. HSD17B7 catalyzes the conversion of zymosterone to zymosterol. DHCR24 catalyzes the conversion of desmosterol to cholesterol. EBP catalyzes the conversion of zymostenol into lathosterol. SC5D catalyzes the conversion of lathosterol into 7-dehydrocholesterol. DHCR7 catalyzes the conversion of 7-dehydrocholesterol to form cholesterol and is the final step in cholesterol synthesis. IDI1/2 is the cytoplasmic enzyme involved in cholesterol synthesis 8, 32-35. Taken together, many genes play a central role in cholesterol synthesis, including HMGCR, SQLE, HMGCS1, FDFT1, LSS, MVK, PMK, MVD, FDPS, CYP51, TM7SF2, LBR, MSMO1, NSDHL, HSD17B7, DHCR24, EBP, SC5D, DHCR7, IDI1/2 (Fig. 1).

## The potential of cholesterol synthesis genes in drug development

As described above, the cholesterol synthesis pathway involves multiple genes. Especially, HMGCR and SQLE are the rate-limiting enzymes in cholesterol synthesis. Statins have been widely used for the treatment of ASCVD by suppressing HMGCR 36-38. Many studies have shown that statins increase survival rates for cancer patients, including prostate cancer (PCa), lung cancer, gastric cancer (GC), renal cell carcinoma (RCC), breast cancer, colorectal cancer, ovarian cancer, pancreatic cancer, esophageal cancer, endometrial cancer, suggesting that HMGCR is a broad-spectrum anticancer and cardiovascular disease target 39, 40. Many drugs have entered the stage of market or clinical trials by targeting other cholesterol synthesis genes, such as SQLE, FDFT1, LSS, FDPS, CYP51, and EBP (Table 1). The SQLE inhibitors include terbinafine 41, 42, liranaftate 43, naftifine 44, Butenafine Hydrochloride 44, Amorolfine Hydrochloride 45, 46. The FDFT1 inhibitors include BPH-652 (also named BMS-188745), S-BPH-652 (also named BMS-188494 or SQ-32709) 47-50, Lapaquistat acetate (also named TAK-475) 51, 52. The LSS inhibitors include Oxiconazole Nitrate (also named Ro 13-8996) 53, 54 and BIBB-515 (also named BIBB 515 BS) 55. The FDPS inhibitors include alendronate 56, incadronate (INC, also named cimadronate or YM-175) 56, 57, ibandronate 56, 58, 59, minodronate 56, risedronate 56, pamidronate 56, zoledronate 56. The CYP51 inhibitors include albaconazole (also named stiefel or UR-9825) 60, 61, arasertaconazole nitrate 62, 63, Bifonazole (also named B3LYP) 64-67, butoconazole (BTZ) 68, 69, clotrimazole 70, dapaconazole 71, 72, eberconazole (EBZ) 73, econazole (also named EcoNai™, SEPA, Spectazole, Ecostatin, or Pevaryl) 74, 75, efinaconazole (also named KP-103 or Jublia) 41, 76, 77, fluconazole 78, flutrimazole 79, 80, fosravuconazole (F-RVCZ, the prodrug of ravuconazole (also named E1224)) 81-86, genaconazole (also named SCH 39304, a racemic mixture that contains 50% of the SCH 42427 and 50% of SCH 42426 enantiomers) 87, 88, HCP002 (a phosphate-modified derivative of voriconazole) 89, IDP113 90, isavuconazole (ISA, the prodrug of isavuconazole (BAL 4815)) 91, 92, ketoconazole (KTC) 93-95, levoketoconazole 95, 96, itraconazole 97, luliconazole 85, 98, miconazole 99, opelconazole (also named PC945) 100, 101, oteseconazole (also named VT-1161) 102, posaconazole 84, 103, pramiconazole (also named R126638) 104-107, quilseconazole (also named VT-1129) 108, 109, SSY726 110, 111, voriconazole (VRC) 112, 113, VT-1598 114, 115. The EBP inhibitors include DSP-0390 (also named RB55ZW48XG) 116-118. Thus, EBP, FDFT1, FDPS, HMGCR, LSS, and SQLE are promising targets for drug development.

## The potential role and mechanism of the circRNA/miRNA axis in cholesterol synthesis

### CircRNA/miR-140-3p/HMGCR and HMGCS1 axis

#### CircRNA_ABCA1

CircRNA_ABCA1 (also named circRNA_36781) is located in the exonic of ABCA1. CircRNA_ABCA1 expression was increased in aortic vessels of HFD-induced apoE-/- mice and H2O2-induced mouse aortic endothelial cells (MAECs) injury model, suggesting that circRNA_ABCA1 is a potential diagnostic biomarker for atherosclerosis. CircRNA_ABCA1 could serve as miR-140-3p sponge increasing vascular endothelial injury and atherosclerosis by regulating the miR-140-3p/MAP2K6 axis 119. MiR-140-3p also suppressed cholesterol biosynthesis by binding and suppressing the 3'UTR of HMGCR and HMGCS1 120, suggesting that circRNA_ABCA1 promoted cholesterol biosynthesis by regulating miR-140-3p/HMGCR and HMGCS1 axis. It is worth mentioning that ABCA1 promotes cholesterol efflux to apolipoprotein A-I (apoA-I) to suppress foam cell formation. Previous studies from our laboratory and others have shown that ABCA1 promoted cholesterol efflux to suppress foam cell formation and atherosclerosis development 121-125. However, the role of circRNA_ABCA1 on ABCA1 expression and cholesterol efflux remains unclear.

#### CircUGGT2 and circ-PRKCH

CircRNA UDP-glucose glycoprotein glucosyltransferase 2 (circUGGT2, also named hsa_circ_0008274) and circ-protein kinase C eta (circ-PRKCH, also named hsa_circ_0032131) are located in the exonic of UGGT2 and PRKCH (encodes PKCη). UGGT2 is the central hub of the endoplasmic reticulum mate network and regulates the PERK-ATF4-CHOP pathway and IL-8 expression 126. PRKCH is a member of the PKC family and regulates RGS2, ABCA1, and CTLA-4 expression. Both UGGT2 and PRKCH play an essential role in lipid metabolism and inflammatory response 127-129. CircUGGT2 and circ-circ-PRKCH could serve as the miR-140-3p sponge 130-132, suggesting that circUGGT2 and circ-PRKCH promoted cholesterol biosynthesis by regulating miR-140-3p/HMGCR and HMGCS1 axis. CircUGGT2 also increased cholesterol efflux by stimulating ABCG1, SR-B1, and miR-186-3p/ABCA1 axis in THP-1 macrophage-derived foam cells 133, suggesting that circUGGT2 not only increased cholesterol synthesis but also cholesterol efflux. Notably, astaxanthin increased the expression of circUGGT2 and then increased cholesterol efflux by stimulating ABCA1, ABCG1, and SR-B1 expression in THP-1 macrophage-derived foam cells. Astaxanthin also suppressed foam cell formation and atherosclerosis development by enhancing ABCA1, ABCG1, and SR-B1 expression in apoE^-/-^ mice 133, 134, suggesting that circUGGT2 may be an anti-atherosclerotic RNA *in vivo*. However, more studies are needed.

### CircRNA/miR-133b and miR-221-5p/SQLE axis

#### CircRNA/miR-133b/SQLE axis

Zeste homolog 2 (EZH2) could encode circRNAs, including circEZH2 (also named hsa_circ_0006357) and hsa_circ_0008324. Many studies have shown that EZH2 plays a crucial role in cholesterol synthesis and atherosclerosis development. EZH2 siRNA and inhibitors promoted cholesterol synthesis by enhancing multiple genes expression, including HMGCS1, FDFT1, SQLE, LSS, CYP51A1, DHCR7, DHCR24, and HMGCR 135, suggesting that EZH2 suppressed cholesterol synthesis. However, EZH2 promoted atherosclerosis development *in vivo*. Specifically, myeloid EZH2 deficiency reduced atherosclerosis development by reducing neutrophil migration and macrophage foam cell inflammatory responses, such as nitric oxide (NO), IL-6, and IL-12 136. EZH2 reduced ABCA1 expression by promoting triple methylation of lysine 27 (H3K27) in the ABCA1 promoter region and then reduced cholesterol efflux to promote foam cell formation and atherosclerosis development 137, 138. EZH2 regulated miR-139-5p methylation and its target STAT1 expression through H3K27me3 and then promoted ox-LDL-induced HASMCs apoptosis, plaque formation, and inflammatory response in atherosclerosis mice 139. EZH2 promoted the expression of MMP2 and MMP9 and their-mediated migration of aortic smooth muscle cells (MASMCs) and atherosclerosis development by promoting the methylation of TIMP2 140. As mentioned above, EZH2 is a parental gene of circEZH2 141, 142. suggesting that circEZH2 may regulate cholesterol synthesis and atherosclerosis development by regulating EZH2 expression. However, more studies are needed.

It is worth noting that circEZH2 could serve as a sponge of miR-133b 142. MiR-133b suppressed SQLE expression by targeting SQLE 3'UTR 143, 144, suggesting that circEZH2 promoted cholesterol synthesis by regulating the miR-133b/SQLE axis. In addition, circEZH2 promoted fatty acid uptake by regulating miR-378b/CD36 and the LPL axis. CircEZH2 also promoted fatty acid uptake by promoting Fatty acid desaturase 1 (FADS1) and stearoyl-CoA desaturase 1 (SCD1) expression 145. Many studies have shown that CD36, LPL, FADS1, and SCD1 promoted atherosclerosis development by regulating lipid metabolism. CD36 promoted cholesterol uptake, foam cell formation, and fatty acid uptake. LPL is responsible for the hydrolysis of triglycerides to glycerol and free fatty acids and is a critical factor in fatty acid uptake. FADS1 and SCD1 mainly promoted unsaturated fatty acid synthesis. Therefore, circEZH2 promoted cholesterol synthesis and uptake to foam cell formation and atherosclerosis development by regulating the miR-133b/SQLE axis and miR-378b/CD36 axis. Indeed, many circRNAs could serve as a sponge of miR-133b, including circ_0005273 146, circRAB3IP 147, circ_0007031 148, circ_0006459 149, circ-HECTD1 150, circ_0039569 151, circ_BIRC6_001271 152, suggesting that these circRNAs promoted cholesterol synthesis by regulating miR-133b/SQLE axis. However, more studies are needed.

#### CircRNAs/miR-221-5p/SQLE axis

Sterol regulatory element binding protein (SREBP) cleavage activating protein (SCAP) could encode circRNAs, including circRNA-SCAP (also named circSCAP, hsa_circ_0001292), has_circRNA_103352, hsa_circ_0065214, hsa_circ_0007291. These circRNAs are located in the exonic of SCAP. SCAP also regulated cholesterol synthesis. SCAP could bind to SREBPs and form SCAP-SREBP complex. When cholesterol in the endoplasmic reticulum (ER) is too low (below 5 %), SCAP binds to the Coat Protein complex II (COPII) protein and escorts the SCAP-SREBP complex from the ER to the Golgi. After several conformational changes, SREBP2 separates from the SCAP-SREBP2 complex and enters the nucleus. SREBPs promoted cholesterol synthesis genes by binding to HMGCR and SQLE 153. As mentioned above, circRNA-SCAP is located in SCAP, suggesting that circRNA-SCAP may regulate cholesterol synthesis by regulating SCAP expression and SCAP-SREBP2 complex.

It is worth noting that circRNA-SCAP may be a potential biomarker of atherosclerotic plaque stability. Serum circRNA-SCAP and phosphodiesterase 3B (PDE3B) were upregulated in 25 patients with cerebral atherosclerosis, and ox-LDL-disposed THP-1 foam cells, whereas miR-221-5p level was decreased. CircRNA-SCAP is a miR-221-5p sponge 154. MiR-221-5p could decrease cholesterol content in the liver by targeting and suppressing SQLE 155, suggesting that circRNA-SCAP promoted cholesterol synthesis by regulating the miR-221-5p/SQLE axis. MiR-221-5p also suppressed PDE3B expression by targeting PDE3B 3'UTR. By regulating the miR-221-5p/PDE3B axis, circRNA-SCAP promoted lipid deposition (total cholesterol (TC) and triglycerides (TG)), apoptosis (increased pro-apoptotic molecule Bax and cleaved-caspase 3 (caspase 3) and decreased anti-apoptotic molecule Bcl-2), inflammation (IL-6, IL-1β, TNFα, and COX-2), and oxidative stress (increased pro-oxidation molecule ROS and malondialdehyde (MDA) level and decreased anti-oxidation molecule superoxide dismutase (SOD) level) 154. Thus, circRNA-SCAP promoted atherosclerosis development by regulating miR-221-5p/SQLE and PDE3B axis. In addition, circRNA-XPO4 also served as a miR-221-5p sponge 156, suggesting that circRNA-XPO4 promoted cholesterol synthesis by regulating the miR-221-5p/SQLE axis. However, more studies are needed.

### CircRNAs/miR-188-5p/HMGCS1 axis

Circ_0001513 increased HMGCS1 expression by serving as a sponge of miR‑188‑5p 157, suggesting that circ_0001513 increased cholesterol synthesis by regulating the miR-188-5p/HMGCS1 axis. In addition, circ-PRMT5 158 and hsa-circRNA-005843 159 could also serve as a sponge of miR‑188‑5p, suggesting that these circRNAs increased cholesterol synthesis by regulating miR-188-5p/HMGCS1 axis. In addition, circ-PRMT5 also serves as a sponge for miR-203 160 and miR-377 161. MiR-203 suppressed atherosclerotic plaque formation by binding and suppressing E26 oncogene homolog 2 (Ets2) expression, which promotes intraplaque proinflammatory phenotype 162. MiR-377 suppressed atherosclerosis development by regulating DNA Methyltransferase 1 (DNMT1)/LPL/GPIHBP1 axis (triglyceride metabolism) and spleen tyrosine kinase (Syk) expression in apoE^-/-^ mice 163, 164. Circ-PRMT5 may promote cholesterol synthesis, intraplaque proinflammatory phenotype, and triglyceride metabolism by regulating the miR-188-5p/HMGCS1 axis, miR-203/Ets2 axis, miR-377/DNMT1/LPL/GPIHBP1 axis, and miR-377/Syk axis. However, circ-PRMT5 could also serve as a sponge of miR-145 165. MiR-145 reduced ABCA1 expression and cholesterol efflux to promote foam cell formation and atherosclerosis development by targeting the ABCA1 3'UTR 125, 166. Therefore, miR-145/ABCA1 axis may attenuate the pro-atherogenic effect of circ-PRMT5. More studies are needed to confirm the role of circ-PRMT5 on atherosclerosis *in vivo*.

### CircRNAs/miR-34a-5p/ACSL1 axis and miR-141-3p/YWHAG and PTEN axis

HMGCS1 could endcode five circRNAs, including circ-HMGCS1 (also named circHMGCS1, hsa_circ_0072391), hsa_circ_0072387, circHMGCS1-016 (also named hsa_circ_0008621), hsa_circ_0072389, hsa_circ_0072386. These circRNAs are located in the exon 4-6 of HMGCS1, and serve as a sponge of miR‑338‑5p 167. However, the role of miR-338-5p in atherosclerosis has unclear. Interestingly, circ-HMGCS1 and hsa_circ_0072387 suppress lipid synthesis. CircHMGCS1-016 could increase CD73 and galectin (GAL-8) expression by serving as a sponge of miR-1236-3p 168. CD73 has a weak anti-atherosclerosis effect in the early stages of the disease. However, as the disease progresses, CD73 promotes the accretion of atherosclerotic plaque by suppressing lipid catabolism 169. GAL-8 promotes atherosclerosis development by enhancing inflammation, platelet aggregation, and thromboxane generation 170. Therefore, circHMGCS1-016 may promote atherosclerosis by regulating miR-1236-3p/CD73 and the GAL-8 axis. The role of hsa_circ_0072389, and hsa_circ_0072386 in lipid synthesis and atherosclerosis has unclear. More studies are needed.

#### CircRNAs/miR-34a-5p/ACSL1 axis

Circ-HMGCS1 could serve as a sponge of miR‑34a‑5p 171, miR-581 172, miR-892a 172, and miR-503-5p 173. MiR-34a-5p suppressed long-chain acyl-CoA synthetase 1 (ACSL1) expression by targeting ACSL1 3'UTR, an essential enzyme for the synthesis of fatty acyl-CoA, triglycerides, phospholipids, and cholesterol esters 174, 175. However, ACSL1 also promotes lipid efflux. MiR-34a-5p increases the level of triglycerides and cholesterol in the liver by suppressing ACSL1 expression 175, suggesting that circ-HMGCS1 may suppress lipid levels although it inhibits lipid synthesis by regulating miR-34a-5p/ACSL1 axis. In addition, miR-34a-5p also increased lipid droplet accumulation by suppressing adipose triglyceride lipase (ATGL) expression which is a key lipolysis gene and enhances adipose tissue lipolysis 176. MiR-34a-5p suppressed ADAM10 expression by targeting ADAM10 3'UTR 177. MiR-581 suppressed ABCG1 expression by targeting ABCG1 3'UTR 178. Many studies have shown that ADAM10 and ABCG1 play a key role in promoting cholesterol efflux, suggesting that circ-HMGCS1 suppressed lipid accumulation by regulating miR-34a-5p/ACSL1, ATGL, ADAM10 axis, and miR-581/ABCG1 axis. MiR-503-5p promoted proinflammatory cytokines and adhesion molecules level and atherosclerosis development by regulating smad family members 1 (smurf1), 2 (smurf2), and 7 (Smad7) in RAW264.7 macrophage-derived foam cells and apoE^-/-^ mice 179, suggesting that circ-HMGCS1 may suppress proinflammatory cytokines by regulating miR-503-5p/smurf1, smurf2, Smad7 axis. Therefore, circ-HMGCS1 promotes lipid synthesis by regulating the miR-34a-5p/ACSL1 axis. Circ-HMGCS1 suppresses lipid accumulation and proinflammatory cytokines by regulating miR-34a-5p/ACSL1, ATGL, ADAM10 axis, miR-581/ABCG1 axis, and miR-503-5p/smurf1, smurf2, Smad7 axis. Circ-HMGCS1 may be an anti-atherosclerotic RNA. However, more studies are needed.

Notable, many circRNAs could serve as a sponge of miR-34a-5p, including circOgdh (also named mmu_circ_0000231) 176, circMED12L 180, circ_FURIN 181, circ_CSNK1E 182, circ0036602 183, circ-LRP1B 184, circHUWE1 185, circITGA7 186, circNFIX 187, 188, circRNA-CIDN 189, circ_0009910 190, circ_0039569 191, hsa_circ_0018069 192, suggesting that these circRNAs may promote lipid synthesis but suppress lipid accumulation by regulating miR-34a-5p/ACSL, ATGL, and ADAM10 axis.

#### CircRNAs/miR-141-3p/YWHAG and PTEN axis

Tyrosine 3-monooxygenase/tryptophan 5-monooxygenase activation protein gamma (YWHAG, encoding 14-3-3γ) regulates lipid metabolism and glucose homeostasis by regulating the localization of Lipin1 and GLUT4 193, 194. PTEN also regulates lipid metabolism and glucose homeostasis by regulating SREBP-1c and GSK-3β expression 195. Hsa_circ_0072387 could serve as a sponge of miR-141-3p (also named miR-141) and miR-503-5p 196, 197. MiR-141-3p increases triglyceride and cholesterol synthesis by upregulating YWHAG and downregulating PTEN expression, respectively 198, suggesting that hsa_circ_0072387 may suppress lipid synthesis by regulating miR-141-3p/YWHAG and PTEN axis. As mentioned above, miR-503-5p promoted proinflammatory response and atherosclerosis development by regulating smurf1, smurf2, and Smad7; thus, hsa_circ_0072387 may suppress lipid synthesis and pro-inflammatory response by regulating miR-141-3p/YWHAG and PTEN axis, miR-503-5p/smurf1, smurf2, Smad7 axis.

Many circRNAs could serve as a sponge of miR-141-3p, including circDLG1 199, circDIDO1 200, circ_100395 (also named exo-circ_100395) 201, circ_0075943 201, circTRPS1 (also named hsa_circ_0085361) 202, circRNA_100338 203-205, circKEAP1 206, circ-LRP6 207, circZEB1 208, circRNA-SMG1.72 (also named circ-SMG1.72) 209, circSOBP 210, hsa_circRNA_100395 211, circ_0061140 212, circATRNL1 213, circ-GBR10 214, suggesting that these circRNAs suppress lipid synthesis by regulating miR-141-3p/YWHAG and PTEN axis.

#### CircRNAs/miR-494-3p/PTEN axis

CircCYP51 (also named circ_0081001) is derived from CYP51 and is a potential biomarker for the diagnosis and prognosis of osteosarcoma (OS) 215. CircCYP51 could serve as a sponge of miR-494-3p 216. MiR-494-3p promoted proinflammatory macrophage polarization by suppressing Wnt signaling in atherosclerosis 217. MiR-494-3p also promoted plasma cholesterol levels by suppressing PTEN 218, 219. As mentioned above, PTEN was negatively correlated with cholesterol synthesis, suggesting that circCYP51 suppresses proinflammatory macrophage polarization and cholesterol synthesis by regulating miR-494-3p/Wnt and PTEN axis.

### CircRNAs/miR-892b and miR-217-5p/DHCR24 axis

#### CircRNAs/miR-892b/DHCR24 axis

CircPTK2 (also named hsa_circ_0003221) is located in exons 3-7 of protein tyrosine kinase 2 (PTK2). CircPTK2 increased DHCR24 expression by serving as a sponge of miR-892b 220, suggesting that circPTK2 promotes cholesterol synthesis by regulating the miR-892b/DHCR24 axis. CircPTK2 could serve as a sponge of miR-1278 221, miR-139-3p 222, and miR-758-3p (miR-758) 223, MiR-1278 suppressed cardiomyocyte inflammation in myocardial ischemia by reducing IL-22 and CXCL14 expression 224, suggesting that circPTK2 promotes cholesterol synthesis and inflammation by regulating the miR-892b/DHCR24 axis and miR-1278/IL-22 and CXCL14 axis. However, miR-758-3p suppressed cholesterol efflux and foam cell formation by targeting ABCA1 3'UTR 225. MiR-758-3p/ABCA1 axis may attenuate the pro-atherogenic effect of circPTK2. More studies are needed to confirm the role of circPTK2 on atherosclerosis *in vivo*.

#### CircRNAs/miR-217-5p/KLF5/DHCR24 axis

CircEZH2 enhanced Krüppel-like factor 5 (KLF5) expression by sponging with miR-217-5p 226. Interestingly, KLF5 increases cholesterol synthesis by activating the DHCR24 promoter 227. As mentioned earlier, circEZH2 promoted cholesterol synthesis and uptake by regulating the miR-133b/SQLE axis and miR-378b/CD36 axis. Therefore, circEZH2 promoted cholesterol synthesis and uptake to enhance foam cell formation and atherosclerosis development by regulating the miR-217-5p/KLF5/DHCR24 axis, miR-133b/SQLE axis, and miR-378b/CD36 axis.

In addition, many circRNAs could serve as a sponge of miR-217-5p, including circROBO1 228, circ_0033596 229, and circ_0002099 230, suggesting that these circRNAs promoted cholesterol synthesis by regulating miR-217-5p/KLF5/DHCR24 axis.

### CircRNAs/miR-122/SREBP-2, HMGCR, and PMK axis

MiR-122 antagonism decreases hepatic lipid metabolism and cholesterol biosynthesis by suppressing several genes expression, including acetyl-CoA carboxylase alpha (ACC1), acetyl-CoA carboxylase beta (ACC2), ATP citrate lyase (ACLY), SCD1, Fatty acid synthase (FASN, also named FAS), SREBP-2, HMGCR, and PMK 10. MiR-122 antagonism is a promising strategy for the treatment of ASCVD. Many circRNAs could serve as a sponge of miR-122, including ciRS-122 (also named hsa_circ_0005963) 231, circRNA_002581 232, circCDK17 233, circ_0007142 234, circ_0011269 235, circ-IARS 236, circ_0072995 237, circFOXO3 (also named hsa_circ_0006404) 238, circ_pleiotrophin (circ_PTN) 239, and circ_1639 240, suggesting that these circRNAs may suppress cholesterol biosynthesis by serving as a sponge of miR-122 (Table 2). Significantly, inhibits miR-122 with LNA-antagomiR-122 (also named SPC3649 or Miravirsen, was developed by SantarisPharma) and N-acetylgalactosamine-conjugated anti-microRNA-122 oligonucleotide (also named RG-101 was developed by Regulus Therapeutics) for the treatment of hepatitis C virus (HCV) infections has completed the phase II trial 241, 242. More importantly, circFOXO3 is located in exon 3 of forkhead box O3 (FOXO3). CircFOXO3 rs12196996, a polymorphism at the gene flanking intron, is associated with circFOXO3 levels and the risk of ASCVD in the Chinese Han population 243. The clinical application potential of circFOXO3 in tumor diagnosis and treatment is immense 244, suggesting that circFOXO3 may be a promising future target in the diagnosis and treatment of cancer and cardiovascular disease.

## Conclusions and Future Directions

Many genes play a central role in cholesterol synthesis, including CYP51, DHCR7, DHCR24, EBP, FDFT1, FDPS, HMGCR, HMGCS1, HSD17B7, IDI1/2, LBR, LSS, MSMO1, MVD, MVK, NSDHL, PMK, SC5D, SQLE, and TM7SF2. Many circRNAs regulate cholesterol synthesis by regulating ACSL1, DHCR24, HMGCR, HMGCS1, PTEN, SQLE, and YWHAG expression by sponging miRNAs. Some circRNAs were also involved in other atherosclerotic risk factors (Table 3). Notable, CYP51, EBP, FDFT1, FDPS, HMGCR, LSS, and SQLE, are promising therapeutic targets for drug development due to many specific inhibitors have been approved and entered into clinical research by targeting these genes. Many circRNAs regulated cholesterol biosynthesis by regulating HMGCR expression via sponging miR-122. Several drugs targeting miR-122 have completed the phase II trial for the treatment of HCV infections, including Miravirsen and RG-101. Thus, the circRNA/miR-122/HMGCR axis is a promising therapeutic axis for drug development. However, several interesting and critical tasks remain to be explored: (1) The naming of circRNA is not uniform and even a little confusing, such as HMGCS1 could encode five circRNA, including hsa_circ_0072391, hsa_circ_0072387, hsa_circ_0008621, hsa_circ_0072389, and hsa_circ_0072386. However, hsa_circ_0072391 is also named circ-HMGCS1 or circHMGCS1, while hsa_circ_0008621 is also named circHMGCS1-016. SCAP could encode circRNAs, including hsa_circ_0001292, has_circRNA_103352, hsa_circ_0065214, and hsa_circ_0007291. However, only hsa_circ_0001292 is also named circRNA-SCAP or circSCAP. (2) Several circRNAs not only promoted cholesterol biosynthesis but also promoted cholesterol efflux or suppressed proinflammatory cytokines, including circUGGT2, circ-PRMT5, circ-HMGCS1, circOgdh, circMED12L, circ_FURIN, circ_CSNK1E, circ0036602, circ-LRP1B, circHUWE1, circITGA7, circNFIX, circRNA-CIDN, circ_0009910, circ_0039569, hsa_circ_0018069, and circPTK2. The role of these circRNAs in atherosclerosis remains to be investigated *in vivo*. (3) The state of the disease may affect circRNAs studies, such as circHMGCS1-016. CircHMGCS1-016 may exhibit an anti-atherogenic effect in the early stages of the disease. However, as the disease progresses, circHMGCS1-016 may exhibit a pro-atherogenic effect. The development of drugs and diagnostic reagents must consider the state of disease progression. (4) CircRNAs regulate gene expression through various mechanisms, including sponge miRNA, protein scaffold and sponge, encoding protein, and regulation of splicing and transcription. However, so far, almost all circRNAs regulate cholesterol synthesis genes through sponge miRNA. Whether there are other mechanisms is not clear. (5) Until now, most circRNA's role in cholesterol synthesis has been studied *in vitro*. However, there are many factors influencing the development of the disease. The effect of circRNAs on the disease still needs to be studied *in vivo*. (6) Given that inhibits miR-122 completed the phase II trial, circFOXO3 is a promising target for drug research by sponging miR-122. However, more studies are needed. (7) Many drugs have been approved for market by targeting HMGCR and SQLE expression. Several circRNAs may be promising therapeutic targets for drug development by targeting HMGCR and SQLEM, such as circRNA_ABCA1, circ-PRKCH, circEZH2, and circRNA-SCAP. However, more studies are needed. (8) The development of new drugs usually requires preclinical studies in multiple animal models before clinical application to improve drug development's success rate. The development of circRNAs drugs also requires much research. (9) Current methods of circRNA synthesis are limited by low cyclization efficiency and the high cost of enzymes and other reagents. There is an urgent need to address these issues. (10) The current study has shown that circRNAs have many targets. However, it may be caused by different dosing doses. Whether there are multiple targets *in vivo* still needs much research.

In summary, drugs that target CYP51, EBP, FDFT1, FDPS, HMGCR, LSS, SQLE, and miR-122 have entered the stage of market or clinical trials. CircRNA_ABCA1, circ-PRKCH, circEZH2, circRNA-SCAP, and circFOXO3 are promising therapeutic targets for drug development, specifically circFOXO3. With the progress of science and technology, the deepening of research, and the cooperation of scientific research, we believe there will be the clinical application of circRNAs agents soon.

## Figures and Tables

**Table 1:** The drugs in the market and clinical trials targeting cholesterol synthesis genes.

Name	Structure	Target	Diseases	Status	Refs
Atorvastatin	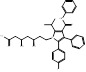 PubChem CID: 60823	HMGCR	ASCVD	Market	36-38
Fluvastatin	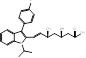 PubChem CID: 446155	HMGCR	ASCVD	Market	36-38
Lovastatin	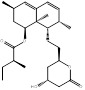 PubChem CID: 53232	HMGCR	ASCVD	Market	36-38
Pravastatin	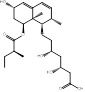 PubChem CID: 54687	HMGCR	ASCVD	Market	36-38
Rosuvastatin	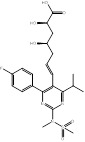 PubChem CID: 446157	HMGCR	ASCVD	Market	36-38
Simvastatin	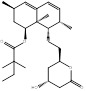 PubChem CID: 54454	HMGCR	ASCVD	Market	36-38
Terbinafine	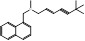 PubChem CID: 1549008.	SQLE	Onychomycosis and superficial dermatomycoses	Market	41, 42
Liranaftate	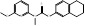 PubChem CID: 3936.	SQLE	Tinea	Market	43
Naftifine	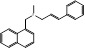 PubChem CID: 47641.	SQLE	Tinea corporis, Tinea cruris, Tinea pedis	Market	44
Butenafine	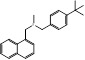 PubChem CID: 2484.	SQLE	Mycoses, onychomycosis, pityriasis versicolor, Tinea corporis, Tinea cruris, Tinea pedis	Market	44
Amorolfine	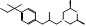 PubChem CID: 54260.	SQLE	Onychomycosis and various local dermal mycoses	Market	45, 46
BPH-652	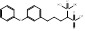 PubChem CID: 10004539.	FDFT1	Cholesterol-lowering agent	early clinical trials (Completed)	47-49
S-BPH-652	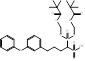 PubChem CID: 154098.	FDFT1	Hyperlipidaemia	Phase 2 (Discontinued)	48-50
Lapaquistat Acetate	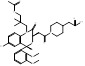 PubChem CID: 9874248.	FDFT1	Hypercholesterolemia	Phase 3 (Completed)	51, 52
Oxiconazole Nitrate	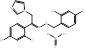 PubChem CID: 9556529.	LSS	Tinea pedis, tinea cruris, and tinea corporis	Market	53, 54
BIBB-515	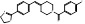 PubChem CID: 501398.	LSS	Hyperlipidemia	Phase 1 (Completed)	NCT02266498 (ClinicalTrials.gov), NCT02266485, 55
Alendronate	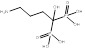 PubChem CID:2088.	FDPS	Corticosteroid-induced osteoporosis; Fracture; Male osteoporosis; Malignant hypercalcaemia; Osteitis deformans; Osteoporosis; Postmenopausal osteoporosis	Market	56
Incadronate	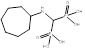 PubChem CID: 3013050.	FDPS	Malignant hypercalcaemia	Market	56, 57
Ibandronate	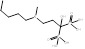 PubChem CID: 6918123.	FDPS	Cancer metastases; Malignant hypercalcaemia; Osteoporosis; Postmenopausal osteoporosis	Market	56, 58, 59
Minodronate	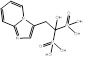 PubChem CID: 130956.	FDPS	Osteoporosis	Market	56
Risedronate	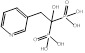 PubChem CID: 5245.	FDPS	Corticosteroid-induced osteoporosis; Male osteoporosis; Osteitis deformans; Osteoporosis; Postmenopausal osteoporosis	Market	56
Pamidronate	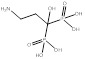 PubChem CID: 4674.	FDPS	Osteoporosis	Market	56
Zoledronate	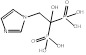 PubChem CID: 68740.	FDPS	Bone metastases; Corticosteroid-induced osteoporosis; Fracture; Male osteoporosis; Malignant hypercalcaemia; Mesothelioma; Multiple myeloma; Osteitis deformans; Postmenopausal osteoporosis	Market	56
Albaconazole	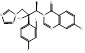 PubChem CID: 208952.	CYP51	Onychomycosis	Phase 2 (Completed)	60, 61
			Candidiasis Vulvaginitis	Phase 2 (Terminated)	NCT00199264
Arasertaconazole nitrate	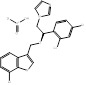 PubChem CID: 9806019	CYP51	Vulvovaginal Candidiasis (VVC)	Phase 3 (Planning)	62, 63
Bifonazole	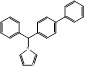 PubChem CID: 2378.	CYP51	Otomycosis, onychomycos, isseborrhoeic dermatitis of the scalp	Market	64-67
Butoconazole	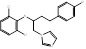 PubChem CID: 47472.	CYP51	Vulvovaginal candidiasis	Market	68, 69
Clotrimazole	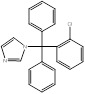 PubChem CID: 2812.	CYP51	Skin, oral and vaginal candida infections	Market	70
Dapaconazole	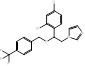 PubChem CID: 51001696.	CYP51	Tinea Pedis	Phase 3 (completed)	NCT03320486, 71, 72
Eberconazole	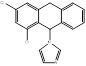 PubChem CID: 72051.	CYP51	Cutaneous fungal infections	Market	73
Econazole	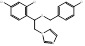 PubChem CID: 3198.	CYP51	Fungal infections such as tinea pedis and cruris, pityriasis versicolor	Market	74, 75
Efinaconazole	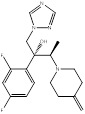 PubChem CID: 489181.	CYP51	Onychomycosis	Market	41, 76, 77
Fluconazole	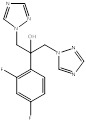 PubChem CID: 3365.	CYP51	Vulvovaginal candidiasis (RVVC)	Market	78
Flutrimazole	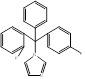 PubChem CID: 3401.	CYP51	Superficial skin fungal infections	Market	79, 80
Fosravuconazole	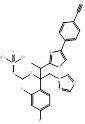 PubChem CID: 9807507.	CYP51	Onychomycosis	Market	81-86
Genaconazole	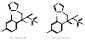 PubChem CID: 452261, 456001.	CYP51	Meningitis, Cryptococcal HIV Infections	Phase 1 (Completed)	NCT00000677, 87, 88
HCP002	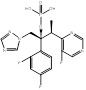 PubChem CID: unknown.	CYP51	Invasive fungal infections (IFI)	Phase 1 (Recruiting)	89
IDP113	Unknown	CYP51	Tinea capitis (Discontinued)	Phase 2 (ongoing on 30 Aug 2010)	90
Isavuconazole	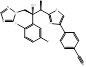 PubChem CID: 6918485.	CYP51	Invasive aspergillosis (IA) and invasive mucormycosis (IM)	Market	91, 92
Ketoconazole	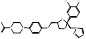 PubChem CID: 456201.	CYP51	Systemic and superficial mycoses, cushing's syndrome (CS)	Market	93-95
Levoketoconazole	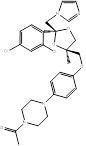 PubChem CID: 47576.	CYP51	CS	Market	95, 96
Itraconazole	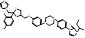 PubChem CID: 55283.	CYP51	Broad spectrum antifungal agent	Market	97
Luliconazole	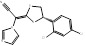 PubChem CID: 3003141.	CYP51	Onychomycosis	Market	85, 98
Miconazole	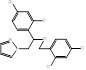 PubChem CID: 4189.	CYP51	Superficial and cutaneous disease	Market	99
Opelconazole	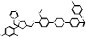 PubChem CID: 121383526.	CYP51	Pulmonary Aspergillosis	Phase 3 (Recruiting)	100, 101
Oteseconazole	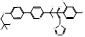 PubChem CID: 77050711.	CYP51	Recurrent Vulvovaginal Candidiasis	Market	102
Posaconazole	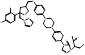 PubChem CID: 468595.	CYP51	Broad-spectrum antifungal	Market	84, 103
Pramiconazole	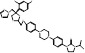 PubChem CID: 3013050.	CYP51	Pityriasis versicolor (PV)	Phase 2 (Completed)	104-107
Quilseconazole	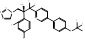 PubChem CID: 91886002.	CYP51	Systemic Cryptococcus infections	Phase 1 (underway)	108, 109
SSY726	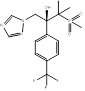 PubChem CID: 486307.	CYP51	Mycoses (Discontinued)	Phase 2	110, 111
Voriconazole	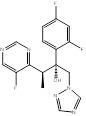 PubChem CID: 71616.	CYP51	IFI	Market	112, 113
VT-1598	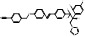 PubChem CID: 126715974.	CYP51	Coccidioidomycosis	Phase 1 (completed)	NCT04208321, 114, 115
DSP-0390	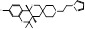 PubChem CID: 154975891.	EBP	Recurrent High-Grade Glioma	Phase 1 (Recruiting)	NCT05023551, 116-118

**Table 2:** The potential role and mechanism of circRNAs in cholesterol synthesis.

CircRNAs	Axis	Refs
circRNA_ABCA1	miR-140-3p/HMGCR and HMGCS1 axis	119, 120
circUGGT2	miR-140-3p/HMGCR and HMGCS1 axis	120, 130, 131
circ-PRKCH	miR-140-3p/HMGCR and HMGCS1 axis	120, 132
circEZH2	miR-133b/SQLE axis	142-144
	miR-217-5p/KLF5/DHCR24 axis	226, 227
circ_0005273	miR-133b/SQLE axis	143, 144, 146
circRAB3IP	miR-133b/SQLE axis	143, 144, 147
circ_0007031	miR-133b/SQLE axis	143, 144, 148
circ_0006459	miR-133b/SQLE axis	143, 144, 149
circ-HECTD1	miR-133b/SQLE axis	143, 144, 150
circ_0039569	miR-133b/SQLE axis	143, 144, 151
circ_BIRC6_001271	miR-133b/SQLE axis	143, 144, 152
circRNA-SCAP	miR-221-5p/SQLE axis	154, 155
circRNA-XPO4	miR-221-5p/SQLE axis	155, 156
circ_0001513	miR-188-5p/HMGCS1 axis	157
circ-PRMT5	miR-188-5p/HMGCS1 axis	157, 158
hsa-circRNA-005843	miR-188-5p/HMGCS1 axis	157, 159
circHMGCS1	miR-34a-5p/ACSL1 axis	171, 175
circOgdh	miR-34a-5p/ACSL1 axis	175, 176
circMED12L	miR-34a-5p/ACSL1 axis	175, 180
circ_FURIN	miR-34a-5p/ACSL1 axis	175, 181
circ_CSNK1E	miR-34a-5p/ACSL1 axis	175, 182
circ0036602	miR-34a-5p/ACSL1 axis	175, 183
circ-LRP1B	miR-34a-5p/ACSL1 axis	175, 184
circHUWE1	miR-34a-5p/ACSL1 axis	175, 185
circITGA7	miR-34a-5p/ACSL1 axis	175, 186
circNFIX	miR-34a-5p/ACSL1 axis	175, 187, 188
circRNA-CIDN	miR-34a-5p/ACSL1 axis	175, 189
circ_0009910	miR-34a-5p/ACSL1 axis	175, 190
circ_0039569	miR-34a-5p/ACSL1 axis	175, 191
hsa_circ_0018069	miR-34a-5p/ACSL1 axis	175, 192
hsa_circ_0072387	miR-141-3p/YWHAG and PTEN axis	196-198
circDLG1	miR-141-3p/YWHAG and PTEN axis	198, 199
circDIDO1	miR-141-3p/YWHAG and PTEN axis	198, 200
circ_100395	miR-141-3p/YWHAG and PTEN axis	198, 201
circ_0075943	miR-141-3p/YWHAG and PTEN axis	198, 201
circTRPS1	miR-141-3p/YWHAG and PTEN axis	198, 202
circRNA_100338	miR-141-3p/YWHAG and PTEN axis	198, 203-205
circKEAP1	miR-141-3p/YWHAG and PTEN axis	198, 206
circ-LRP6	miR-141-3p/YWHAG and PTEN axis	198, 207
circZEB1	miR-141-3p/YWHAG and PTEN axis	198, 208
circRNA-SMG1.72	miR-141-3p/YWHAG and PTEN axis	198, 209
circSOBP	miR-141-3p/YWHAG and PTEN axis	198, 210
hsa_circRNA_100395	miR-141-3p/YWHAG and PTEN axis	198, 211
circ_0061140	miR-141-3p/YWHAG and PTEN axis	198, 212
circATRNL1	miR-141-3p/YWHAG and PTEN axis	198, 213
circ-GBR10	miR-141-3p/YWHAG and PTEN axis	198, 214
circ_0081001	miR-494-3p/PTEN axis	198, 216, 218, 219
circPTK2	miR-892b/DHCR24 axis	220
circROBO1	miR-217-5p/KLF5/DHCR24 axis	226-228
circ_0033596	miR-217-5p/KLF5/DHCR24 axis	226, 227, 229
circ_0002099	miR-217-5p/KLF5/DHCR24 axis	226, 227, 230
ciRS-122	miR-122/SREBP-2, HMGCR, and PMK axis	10, 231
circRNA_002581	miR-122/SREBP-2, HMGCR, and PMK axis	10, 232
circCDK17	miR-122/SREBP-2, HMGCR, and PMK axis	10, 233
circ_0007142	miR-122/SREBP-2, HMGCR, and PMK axis	10, 234
circ_0011269	miR-122/SREBP-2, HMGCR, and PMK axis	10, 235
circ-IARS	miR-122/SREBP-2, HMGCR, and PMK axis	10, 236
circ_0072995	miR-122/SREBP-2, HMGCR, and PMK axis	10, 237
circFOXO3	miR-122/SREBP-2, HMGCR, and PMK axis	10, 238
circ_PTN	miR-122/SREBP-2, HMGCR, and PMK axis	10, 239
circ_1639	miR-122/SREBP-2, HMGCR, and PMK axis	10, 240

**Table 3:** The role and mechanism of circRNAs that are involved in multiple atherosclerotic risk factors.

CircRNAs	Axis	Function	Refs
circRNA_ABCA1	miR-140-3p/HMGCR and HMGCS1 axis	Increased cholesterol synthesis	119, 120
	miR-140-3p/MAP2K6 axis	Increased vascular endothelial injury	119, 120, 130, 131
circUGGT2	miR-140-3p/HMGCR and HMGCS1 axis	Increased cholesterol synthesis	120, 130, 131
	miR-140-3p/MAP2K6 axis	Increased vascular endothelial injury	119, 120, 130, 131
	miR-186-3p/ABCA1 axis	Increased cholesterol efflux	133
circEZH2	miR-133b/SQLE axis	Increased cholesterol synthesis	142-144
	miR-217-5p/KLF5/DHCR24 axis	Increased cholesterol synthesis	226, 227
	miR-378b/CD36 axis	Increased cholesterol uptake	145
	LPL, FADS1 and SCD1	Increased fatty acid uptake	145
circRNA-SCAP	miR-221-5p/SQLE axis	Increased cholesterol synthesis	154, 155
	miR-221-5p/PDE3B axis	Promoted lipid deposition, apoptosis, inflammation, and oxidative stress	154
circRNA-XPO4	miR-221-5p/SQLE axis	Increased cholesterol synthesis	155, 156
	miR-221-5p/PDE3B axis	Promoted lipid deposition, apoptosis, inflammation, and oxidative stress	154-156
circ-PRMT5	miR-188-5p/HMGCS1 axis	Increased cholesterol synthesis	157, 158
	miR-203/Ets2 axis	Increased intraplaque proinflammatory phenotype	162
	miR-377/DNMT1/LPL/GPIHBP1 axis	Increased triglyceride metabolism	161, 163, 164
	miR-145/ABCA1 axis	Increased cholesterol efflux	125, 165, 166
circ-HMGCS1	miR-34a-5p/ACSL1 axis	Promoted lipid synthesis but suppressed lipid accumulation	171, 175
	miR-34a-5p/ATGL axis	Promoted lipolysis	171, 176
	miR-34a-5p/ADAM10 axis	Increased cholesterol efflux	171, 177
	miR-581/ABCG1 axis	Increased cholesterol efflux	172, 178
	miR-503-5p/smurf1, smurf2, Smad7 axis	Suppressed proinflammatory cytokines and adhesion molecules level	173, 179
hsa_circ_0072387	miR-141-3p/YWHAG and PTEN axis	Increased triglyceride and cholesterol synthesis	196, 198
	miR-503-5p/smurf1, smurf2, Smad7 axis	Suppressed proinflammatory cytokines and adhesion molecules level	179, 197
circCYP51	miR-494-3p/PTEN axis	Suppressed cholesterol synthesis	198, 216, 218, 219
	miR-494-3p/Wnt axis	Suppressed proinflammatory macrophage polarization	216, 217
circPTK2	miR-892b/DHCR24 axis	Increased cholesterol synthesis	220
	miR-1278/IL-22 and CXCL14 axis	Promoted cardiomyocytes inflammation	221, 224
	miR-758-3p/ABCA1 axis	Increased cholesterol efflux	223, 225
